# COVID-19, Nutrients and Lifestyle Eating Behaviors: A Narrative Review

**DOI:** 10.3390/diseases12080193

**Published:** 2024-08-22

**Authors:** Giovanni Cangelosi, Sara Morales Palomares, Paola Pantanetti, Alessia De Luca, Federico Biondini, Cuc Thi Thu Nguyen, Stefano Mancin, Marco Sguanci, Fabio Petrelli

**Affiliations:** 1 Unit of Diabetology, ASUR Marche, 63900 Fermo, Italy; giovanni.cangelosi@virgilio.it (G.C.); dr.paolapantanetti@gmail.com (P.P.); 2 Department of Pharmacy, Health and Nutritional Sciences (DFSSN), University of Calabria, 87036 Rende, Italy; sara.morales@unical.it; 3 School of Biosciences and Veterinary Medicine, University of Camerino, 62032 Camerino, Italy; alessia.deluca092@gmail.com; 4 Units of Psychiatry, Ast Fermo, 63900 Fermo, Italy; federco.biondini@gmail.com; 5 Department of Pharmaceutical Administration and Economics, Hanoi University of Pharmacy, Hanoi 100000, Vietnam; cucnguyen.pharm@gmail.com; 6 Humanitas Research Hospital, 20089 Rozzano, Italy; 7 A.O. Polyclinic San Martino Hospital, Largo R. Benzi 10, 16132 Genova, Italy; sguancim@gmail.com; 8 School of Pharmacy, Polo Medicina Sperimentale e Sanità Pubblica “Stefania Scuri”, Via Madonna delle Carceri 9, 62032 Camerino, Italy; fabio.petrelli@unicam.it

**Keywords:** COVID-19, nutrients, lifestyle eating behaviors, narrative review, public health

## Abstract

Background: COVID-19 infection, caused by severe acute respiratory syndrome coronavirus 2 (SARS-CoV-2), quickly emerged as the most significant event of the new millennium. A balanced diet seems to ensure the proper functioning of the immune system and plays a fundamental role in the prevention of viral disease, inflammation, or thrombosis. The principal aim of this secondary study was to investigate the relationship between nutrients, lifestyle eating behaviors, and SARS-CoV-2 infection. Methods: A narrative review was conducted in the PubMed-Medline database, analyzing primary studies. Results: Our review identified 21 relevant studies: 13 focused on vitamins, 1 on omega-3 supplementation, 1 on probiotics, and 6 on lifestyle and dietary behaviors. Vitamin supplementation has shown promise in attenuating COVID-19 symptoms and reducing mortality risk. Specifically, vitamin D has demonstrated efficacy in enhancing immune responses among patients with the disease. While preliminary evidence suggests the potential benefits of omega-3 and probiotic supplementation in improving health outcomes for COVID-19 outpatients, further research is needed to solidify these findings. Conclusions: The lifestyle changes imposed by lockdown measures have adversely affected psychological well-being and exacerbated health issues associated with reduced physical activity and poor dietary habits.

## 1. Introduction

COVID-19 disease, caused by severe acute respiratory syndrome coronavirus 2 (SARS-CoV-2), appeared from the beginning as one of the most tragic events in recent world history [1]. With more than seven hundred million infections and potentially seven million deaths as of 13 April 2024, approximately four years after its global spread, it has reached dimensions that present significant challenges for effective management [1,2]. Acute or chronic malnutrition, along with incorrect lifestyle factors, can lead to a reduced immune response in the body, thereby increasing the risk of infection, particularly in vulnerable populations. This risk was evident from the early moments of the pandemic and was further exacerbated by drastic changes in usual dietary habits [3,4,5].

Diabetes and cardiovascular disease, often accompanied by comorbidities such as obesity, are major risk factors associated with a predisposition to developing severe COVID-19 [6,7,8,9,10,11]. Adipose tissue, in particular, can be considered a reservoir for viruses due to the presence of angiotensin-converting enzyme 2 (ACE-2), which acts as a receptor for SARS-CoV-2. The higher the amount of adipose tissue, the more significant the presence of ACE-2 receptors [12,13,14,15]. Once a viral infection occurs, there is a disruption in the function of memory T cells, which can lead to the destruction of adipose tissue. This mechanism is particularly pronounced in individuals with obesity. Over time, this process contributes to the maintenance of a local or systemic chronic inflammatory state and suppresses immune functions and host defenses [16,17].

A balanced diet and proper lifestyle behaviors appear to ensure the proper functioning of the immune system and may play a fundamental role in the prevention of viral infections, inflammation, and thrombosis. One molecule that seems to play a role against COVID-19 is platelet-activating factor (PAF), which affects the activity of ACE-2 and can be directly influenced by diet [18]. Additionally, we must consider the reduction in physical activity due to the closure of sports facilities and limited daily outings. These conditions contribute to an increased risk of developing various diseases, including obesity and heart disease, which the American Centers for Disease Control and Prevention (CDC) has shown to be associated with a higher risk of severe COVID-19 symptoms [19,20].

Furthermore, the pandemic has underscored the importance of maintaining a healthy lifestyle to bolster the immune system. Regular physical activity, a balanced diet rich in essential nutrients, and the avoidance of harmful habits such as smoking and excessive alcohol consumption are crucial in reducing the risk of severe outcomes from COVID-19. Nutritional interventions, such as ensuring adequate intake of vitamins D and C, zinc, and omega-3 fatty acids, have been suggested to support immune function and mitigate inflammatory responses [21,22]. The role of micronutrients in enhancing immune resilience highlights the need for public health strategies that promote nutritional education and support, particularly for at-risk populations.

The principal aim of this review was to investigate the relationship between nutrients, lifestyle eating behaviors, and SARS-CoV-2 infection.

## 2. Materials and Methods

### 2.1. Study Design

This study employs a narrative review methodology, which aims to offer a qualitative and interpretative synthesis of the literature on the relationship between nutrients, lifestyle eating behaviors, and SARS-CoV-2 infection.

### 2.2. Definition of Research Question

The research questions guiding this narrative review are twofold: (1) Association Between Diet, Nutrients, and Lifestyle Eating Behaviors with COVID-19: Is there an association between diet, specific nutrients, or lifestyle eating behaviors and the incidence, severity, or progression of COVID-19? (2) Role of Nutritional Interventions in COVID-19 Management: Can nutritional interventions play a significant role in the management of COVID-19, potentially influencing outcomes such as infection rates, disease severity, recovery times, and overall patient prognosis?

These questions were developed using the PICOS framework [23] to ensure a structured and comprehensive approach: P (population): adult COVID-19 population; I (intervention): nutrients, dietary support, or lifestyle eating behaviors; C (comparison): nutrients, dietary support, or lifestyle eating behaviors versus different interventions and/or no interventions; O (outcomes): qualitative and quantitative outcomes; S (study design): primary studies. By employing the PICOS framework, this review systematically examines the relationships between dietary factors and COVID-19, evaluating both qualitative and quantitative outcomes to provide robust evidence for public health and clinical practices.

### 2.3. Literature Search and Criteria

The search strategy for this narrative review was conducted using the PubMed-Medline database, aiming to capture a comprehensive range of primary studies published in English during the COVID-19 pandemic. Following the initial search to identify the total number of records, the article screening process was conducted by two academic researchers (GC and SM). In cases of disagreement between the two, a third researcher (MS) was involved to reach a consensus. EndNote 20 (© 2024 Clarivate) was used for the bibliographic management of the analyzed records. The following search strings were used on PubMed (Supplementary Materials File S1: Search Strategy).

### 2.4. Data Synthesis

The selected studies underwent a rigorous, two-stage analysis process. Initially, they were categorized based on several criteria: first author, year/country, nutrients/lifestyle eating behaviors, type of study, sample, principal intervention, principal bias, and principal results. This categorization ensured a structured approach for synthesizing the identified literature. Following this, a comprehensive narrative synthesis was conducted. This synthesis integrated the results from different primary study designs, offering a holistic perspective on the topic while capturing the nuances and intricacies of each individual study. This approach ensured that the wealth of information from the studies was presented cohesively, emphasizing both the commonalities and unique aspects of the researched topic.

## 3. Results

**Figure 1:**
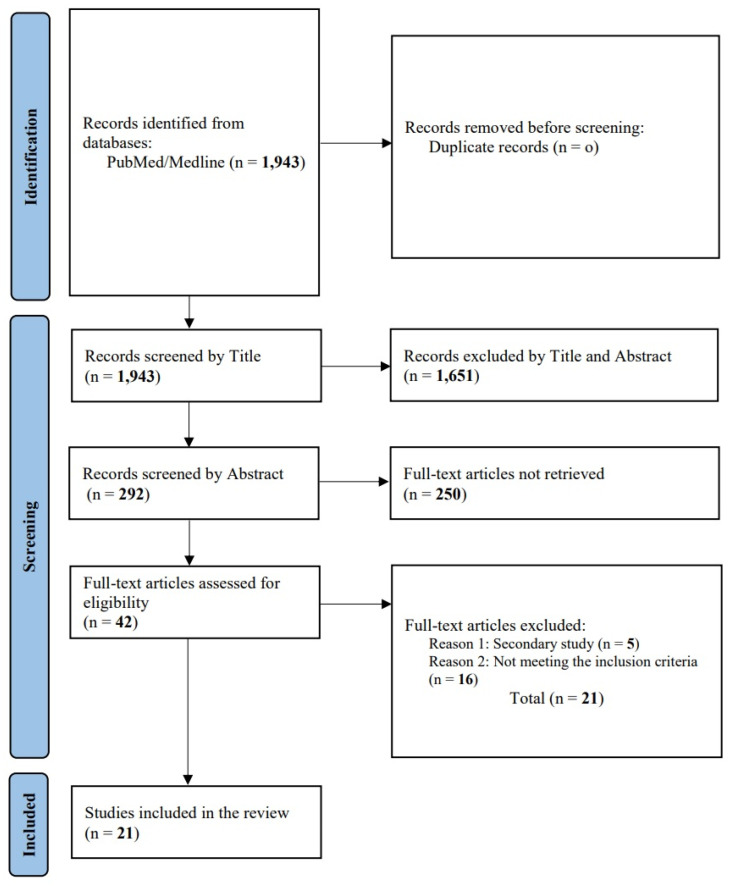
Study selection flow chart.

A search of the PubMed-Medline database yielded a total of 1943 articles. After an initial screening of titles, 1651 articles were excluded. The remaining 292 articles were further screened by analyzing their abstracts, resulting in the removal of 250 articles that were not relevant. Subsequently, 42 full-text articles were evaluated for detailed assessment. Of these, 21 articles were excluded for not meeting the predefined inclusion criteria. Following this comprehensive screening process, a total of 21 studies were ultimately included in this narrative review (Figure 1).

### 3.1. Characteristics of the Studies Included

The majority of the studies included were randomized controlled trials (RCTs), both multi-center [24,25,26,27,28,29] and single-center [30,31,32,33,34,35]. Some studies used a retrospective observational design [36,37], while others adopted a double-blind pilot RCT [38]. Additionally, various cross-sectional studies investigated lifestyle eating behaviors [39,40,41,42,43]. The nutrients and eating behaviors studied are diverse. The studies focused on vitamin D3 [24,25], vitamin C and zinc gluconate [26], vitamin D, magnesium, and vitamin B12 [36], vitamin D [27,28,29,30,31,38,44], multi-vitamins (A, B, C, D, and E) [33], omega-3 [34], probiotics [35], and lifestyle eating behaviors [37,39,40,41,42,43]. In terms of origin, 22% of the studies are from Iran and 17% from Italy, reflecting a high representation of these countries in the research on nutrients and eating behaviors. Eighty-six percent of the studies were conducted in 2020, showing a concentration of research in that specific year. Regarding the type of study, 71% of the included studies are RCTs (both multi-center and single-center), 14% are cross-sectional studies, and 10% are retrospective observational studies (Table 1).

### 3.2. Vitamin D and Other Vitamins

Several studies have examined the impact of vitamin D on COVID-19 patients, demonstrating varied yet insightful outcomes. In a multi-center RCT in Riyadh, Saudi Arabia, 69 patients with mild COVID-19 symptoms were given either 1000 IU or 5000 IU of vitamin D3 daily for 14 days. The 5000 IU group showed significantly faster recovery times for cough (6.2 ± 0.8 days vs. 9.1 ± 0.8 days, *p* = 0.007) and ageusia (11.4 ± 1.0 days vs. 16.9 ± 1.7 days, *p* = 0.035) compared to the 1000 IU group [24]. Similarly, in São Paulo, Brazil, a multi-center RCT with 240 moderate to severe COVID-19 patients who received either a single 200,000 IU dose of vitamin D3 or a placebo found no significant differences in hospital stay duration [7.0 (4.0–10.0) days vs. 7.0 (5.0–13.0) days, *p* = 0.59], hospital mortality [7.6% vs. 5.1%, *p* = 0.43], intensive care unit (ICU) admissions [16.0% vs. 21.2%, *p* = 0.30], mechanical ventilation need [7.6% vs. 14.4%, *p* = 0.09], serum calcium [0.02 mg/dL, *p* > 0.99], creatinine [0.06 mg/dL, *p* > 0.99], and C-reactive protein levels [–0.66 mg/L, *p* = 0.99] between the groups [25]. Furthermore, a retrospective observational study in Singapore explored the effects of a combination of vitamin D3 (1000 IU), magnesium (150 mg/day), and vitamin B12 (500 mcg/day) on 43 COVID-19 patients aged ≥50 years. The experimental group showed a significantly lower requirement for oxygen therapy compared to the control group (3 patients vs. 16, *p* = 0.006). The combination treatment significantly reduced the risk of needing oxygen therapy or ICU admission (OR = 0.13, *p* < 0.05) [36].

A pilot double-blind RCT with 30 healthy elderly males showed that the intervention group, which received 2000 IU/day of vitamin D for six weeks, exhibited an increase in serum creatine kinase levels, indicating a protective effect against muscle catabolism, but no significant improvements in respiratory or general body status [38]. Similarly, another RCT involving 40 symptomatic or mildly symptomatic COVID-19 patients with low vitamin D levels found that the intervention group, which received 60,000 IU of cholecalciferol daily for one week, had significantly higher 25OH-D levels by day 14 and a significant decline in fibrinogen levels (*p* < 0.01) [44].

In Iran, a study with 106 hospitalized COVID-19 patients showed that the intervention group, receiving 25 µg of 25OH-D daily for 30 days, had significantly increased 25OH-D levels and higher lymphocyte counts (*p* = 0.03). Trends toward reduced hospitalization, ICU duration, and mortality were observed, although these were not statistically significant [27]. Moreover, in a multi-center RCT in France with 254 elderly patients, the high-dose vitamin D group (400,000 IU) exhibited a significantly lower 14-day mortality rate (6% vs. 11%; adjusted OR = 0.39, *p* = 0.049), although this effect was not maintained at 28 days [28]. In contrast, a multi-center RCT involving 218 adults hospitalized with mild to moderate COVID-19 found that participants who received a single 500,000 IU dose of vitamin D3 or a placebo showed no significant differences in median hospital stay, ICU admissions, or hospital deaths [29]. An RCT with 50 COVID-19 patients deficient in vitamin D demonstrated that those who received 25,000 IU/day for four days, followed by 25,000 IU/week for six weeks, had significantly shorter hospital stays (4 vs. 8 days; *p* = 0.003) [30]. Similarly, in Russia, another RCT with 129 COVID-19 patients administered 50,000 IU of cholecalciferol on the first and eighth days of hospitalization. This intervention led to significant increases in serum 25OH-D, neutrophil, and lymphocyte counts (*p* = 0.04 and *p* = 0.02), while C-reactive protein levels were significantly lower (*p* = 0.02) [31].

Lastly, a study involving 100 critically ill COVID-19 patients revealed that daily administration of 500 mg of vitamin C for 14 days resulted in significantly lower serum potassium levels (3.93 vs. 4.21 mEq/L, *p* < 0.01) and a higher median survival duration (8 vs. 4 days, *p* < 0.01). More survivors were observed in the vitamin C group (16.1% vs. 2.9%; *p* = 0.028) [32]. Furthermore, another Iranian RCT with 60 ICU-admitted COVID-19 patients tested a combination of vitamins A, D, E, C, and B. This intervention led to significant improvements in serum vitamin levels and a decrease in C-reactive protein (*p* < 0.001) [33].

### 3.3. Omega-3 and Probiotics

Doaei et al. [34] conducted a double-blind RCT in Iran on 101 critically ill COVID-19 patients, comparing omega-3 supplementation to standard care. The treatment group (n = 28) received 1000 mg of omega-3 daily for two weeks, starting 24 h after ICU admission, including 400 mg of eicosapentaenoic acid (EPA) and 200 mg of docosahexaenoic acid (DHA). The intervention group showed a significantly higher 1-month survival rate (21% vs. 3%, *p* = 0.003), improved arterial pH (7.30 vs. 7.26, *p* = 0.01), bicarbonate levels (22.00 vs. 18.17, *p* = 0.01), and base excess (−4.97 vs. −3.59, *p* = 0.01) compared to the control group, with no significant differences in PO2 or PCO2 levels.

In another RCT [35] with 293 symptomatic COVID-19 outpatients, participants were randomized to receive either a probiotic formula for 30 days (147 completed the RCT) or a placebo (146 completed the study). At day 30, 53.1% of the probiotic group achieved complete symptomatic remission and viral clearance compared to 28.1% of the control group (RR: 1.89, 95% CI, 1.40–2.55; *p* < 0.001). There were no hospital or ICU admissions or deaths during the study, and the probiotic group experienced a reduction in the duration of symptoms such as fever, cough, headache, myalgia, shortness of breath, nausea, diarrhea, and abdominal pain compared to the control group.

### 3.4. Lifestyle Eating Behaviors

Studies highlight how COVID-19 restrictions have influenced body weight, physical activity, and dietary habits. During the lockdown, Bhutani et al. [39] observed an average body weight increase of 0.62 kg and an increase in BMI in a sample of 727 U.S. adults. Participants reported increased boredom and stress, reduced appetite control, and a greater desire for salty and processed foods. Similarly, Skotnicka et al. [37] found, in a sample of 1071 European adults, an increase in the frequency of daily meals and consumption of sweet foods, fruits, and alcohol, accompanied by a reduction in physical activity and an increase in BMI in 38.51% of women and 37.34% of men.

Cicero et al. [40], studying 359 Italian subjects, did not find significant changes in BMI but observed that 32% of participants increased their food intake, while 50% did not change their diet. In line with these results, Mascherini et al. [41] reported an increase in body weight and a decrease in work-related physical activity in 1383 people from the University of Florence. However, vigorous exercise increased, while the quality of the diet changed with a higher selection of grains and fruits. Chin et al. [42] found that 1319 Malaysians reported improved eating habits in 41.2% of cases, but a reduction in physical exercise in 36.3% and sleep quality in 25.7%. The percentage of those who lost weight was similar to that of those who gained weight (32.2% and 30.7%). Finally, Paltrinieri et al. [43], analyzing 1826 Italians, highlighted a decrease in physical activity in 35.1% and a deterioration in diet in 7.9%. Both standard and remote work reduced the likelihood of worsening physical habits, with women showing more changes in well-being and nutritional habits compared to men. People aged ≥65 found it more challenging to modify their routine compared to younger individuals.

## 4. Discussion

This review investigated the significant influence of COVID-19 restrictions on physical health and eating behaviors, with a prominent focus on the role of vitamin supplementation. The data reveal complex interactions between lifestyle changes and nutritional interventions during the pandemic. Firstly, the impact of lockdown measures on body weight and activity levels demonstrates a significant increase in weight and BMI, attributed to increased boredom, stress, and changes in eating habits [39]. This phenomenon may also be due to an increased frequency of meals and consumption of sweets, fruits, and alcohol, accompanied by a reduction in physical activity [37,40]. While these short-term lockdown measures are essential to protect susceptible populations from disease, it is crucial to address and mitigate their negative health impacts. Implementing strategies such as promoting home-based physical exercise routines, encouraging healthy eating habits through online nutritional guidance, and providing mental health support can help improve overall health during lockdowns.

An additional critical aspect analyzed is the overall decrease in physical activity, caused by more sedentary working conditions and broader lifestyle changes during the pandemic. These are also identifiable as significant factors in health promotion. These findings are consistent with further studies [45,46,47], which explored the impact of lockdown measures on diet and physical activity, finding a significant reduction in overall physical activity and a concurrent increase in sedentary behaviors. To counteract these trends, public health policies should focus on creating accessible and safe opportunities for physical activity within the constraints of lockdowns, such as virtual fitness classes and outdoor exercise areas that adhere to social distancing guidelines. Moreover, emphasizing the importance of mental well-being and providing resources for coping with stress can enhance the effectiveness of lockdown measures in protecting public health. Both studies emphasize that the decrease in physical activity, combined with less healthy eating habits, may contribute to an increase in body weight and BMI, thereby increasing the risk of metabolic and cardiac diseases. Therefore, it is imperative to balance the disease prevention benefits of short-term lockdowns with proactive measures to promote and maintain physical and mental health, ensuring a holistic approach to public health during such critical times.

Analyzing the potential protective role of vitamins, particularly vitamin D, against complications related to COVID-19, several studies [27,29,30,38] have indicated possible benefits in mitigating muscle catabolism and improving immune responses. Improvements in markers of muscle and immune health, such as serum creatine kinase levels and lymphocyte counts, suggest a significant role of vitamin D in reducing inflammation and supporting immune function. These findings are consistent with recent studies that have explored the potential of this vitamin in counteracting the negative progression and both short- and long-term complications of COVID-19 infection [48]. The pleiotropic functions of vitamin D extend beyond calcium homeostasis and bone health, including critical roles in modulating the immune system, regulating cell proliferation and differentiation, and inhibiting inflammatory responses. Vitamin D exerts its effects through the vitamin D receptor (VDR), present in numerous cell types, including T and B lymphocytes, macrophages, and dendritic cells. These cells are essential for orchestrating an effective immune response [49]. Consequently, as highlighted by previous research [50,51], its benefits may be multifactorial, influencing various physiological processes crucial during a viral infection like COVID-19 with immunomodulatory effects aimed at enhancing body defense mechanisms and potentially reducing the severity of infections. However, it is important to note that studies involving the administration of high doses of vitamin D [25,29] have not demonstrated significant efficacy in managing moderate to severe cases of COVID-19. This suggests that, while vitamin D can support general immune health, its role as a standalone treatment for COVID-19 is limited.

Additionally, the supplementation of omega-3 fatty acids and probiotics may represent a complementary strategy to medical therapy. These supplements have been associated with improvements in respiratory and renal function in critically ill COVID-19 patients [34,35], as well as direct modulation of inflammatory responses and enhancement of intestinal barrier function through their effects on the gut microbiota [52].

Lastly, following the COVID-19 pandemic, it has become clear how crucial it is to adopt targeted public health interventions that promote healthy eating, daily physical activity, and adequate nutritional supplementation. Studies have highlighted not only the negative effects of the restrictions imposed during the pandemic but also the importance of preparing communities to face future health events through a holistic approach to health. Future research should continue to explore the complex mechanisms of nutritional interventions, such as vitamins, probiotics, and omega-3 fatty acids, which have shown potential benefits, as evidenced by studies conducted on other conditions [53,54]. Therefore, it is essential to further educate and raise awareness among the population about the correlation between a balanced diet, regular physical activity, and overall well-being. Promoting active and healthy lifestyles not only enhances individual resilience against diseases but also improves public health outcomes overall.

### Limitations

This study has several limitations. The standardized nutritional measures included in the research were not uniform, and there was a lack of comprehensive meta-analysis of the data. It is important to note that this review is narrative in nature. Unlike systematic reviews, narrative reviews do not typically include a formal assessment of the methodological quality and risk of bias of the included studies. This inherent limitation may affect the reliability and generalizability of the conclusions drawn. Furthermore, the variability in study design complicated direct comparisons between different study groups, given the heterogeneity of populations in terms of factors such as ethnicity, age, and severity of infection. In the RCTs included, group assignment was carried out using a common random sampling method. However, dietary lifestyle studies relied on self-reporting, introducing potential self-reporting biases. Additionally, the omission of treatment for immunodepression among included patients represents a significant limitation, as it could influence outcomes related to immune response. Finally, another significant limitation is the temporal restriction of the studies analyzed, which focused exclusively on the COVID-19 pandemic phase, thereby limiting the generalizability of the results over the long term and across different epidemiological contexts.

## 5. Conclusions

This study investigated the impact of COVID-19 restrictions on physical health, dietary behaviors, and the effectiveness of integrated nutritional strategies. Data analysis revealed complex interactions between lifestyle changes and nutritional interventions during the pandemic, highlighting increased body weight and significant shifts in eating habits. Specifically, the roles of vitamins, probiotics, and omega-3 fatty acids were examined, showing potential benefits in mitigating inflammation, supporting immune function, and enhancing overall health. The effectiveness of these approaches varied depending on the clinical context and individual conditions.

Additionally, the duration of lockdown measures significantly influenced health outcomes. Prolonged lockdowns exacerbated negative health effects such as weight gain and decreased physical activity, whereas shorter lockdowns had comparatively milder impacts. This underscores the importance of considering lockdown duration in public health strategies to balance disease prevention with minimizing adverse health outcomes.

The findings suggest the need for targeted public health interventions that promote healthy eating, regular physical activity, and appropriate nutritional supplementation. These strategies not only enhance resilience against diseases but also improve overall well-being. Future research should explore the mechanisms underlying these interactions to optimize preventive and therapeutic strategies for emerging health challenges.

## Figures and Tables

**Table 1:** Characteristics of the Studies Included.

First Author/Year/Country	Nutrients/Lifestyle Eating Behaviors	Type of Study	Sample	Principal Intervention	Principal Results
Sabico et al. (2020)/Saudi Arabia	Vitamin D3	Multi-center RCT	36 IG, 33 CG	Two weeks oral 5000 IU for IG or 1000 IU for CG	Significantly shorter days needed to resolve cough (6.2 ± 0.8 vs. 9.1 ± 0.8; *p* = 0.007) and ageusia (11.4 ± 1.0 vs. 16.9 ± 1.7; *p* = 0.035)
Murai et al. (2020)/Brazil	Vitamin D3	Multi-center RCT	120 IG, 120 CG	Single oral dose of 200,000 IU of vitamin D3 or placebo	No significant differences found for hospital mortality, ICU admission, and mechanical ventilation support
Thomas et al. (2020)/USA	VIT-C GZ	Multi-center RCT	48 VIT-C, 58 GZ, 58 both, 50 usual care	8000 mg of VIT-C (2–3 times/day with meals) 50 mg of GZ at bedtime, both therapies, or usual care	No significant differences in primary endpoint achievement among groups (*p* = 0.45)
Tan et al. (2020)/Singapore	VIT-D Magnesium and B12	Retrospective cohort	17 DMB IG, 26 CG	1000 IU D3, 150 mg/d magnesium, and 500 mcg/d vitamin B12 orally for 14 days after hospital admission	DMB exposure associated with lower odds of oxygen therapy or ICU support in univariate and multivariate analysis
Caballero-García et al. (2020)/Spain	VIT-D	Pilot double-blind RCT	15 IG, 15 CG	6 weeks of treatment with VIT-D (2000 IU/day)	Increase in serum creatine kinase levels
Rastogi et al. (2020)/India	Cholecalciferol	RCT	16 IG, 24 CG	60,000 IU oral per day of cholecalciferol for 7 days targeting 25OH-D > 50 ng/ml	Mean duration of SARS-CoV-2 negativity similar in both groups (*p* = 0.283)
Maghbooli et al. (2020)/Iran	Cholecalciferol	Multi-center RCT	53 IG (24 completed), 53 CG (19 completed)	25 μg of 25OH-D daily	At 30 and 60 days, higher proportion of sufficient 25OH-D concentration in IG compared to CG
Annweiler et al. (2020)/France	VIT-D	Multi-center RCT	127 high-dose IG, 127 standard-dose CG	High-dose (400,000 IU) and standard-dose (50,000 IU) vitamin D3	No maintained protective effect at 28 days; similar death rates between high-dose and standard-dose groups (*p* = 0.29)
Mariani et al. (2020–2021)/Argentina	VIT-D	Multi-center RCT	115 IG, 103 CG	500,000 IU of vitamin D3 (5 capsules of 100,000 IU)	No significant differences for ICU admissions or in-hospital mortality between groups
De Niet et al. (2020–2021)/Belgium	VIT-D	Single-center RCT	26 IG, 24 CG	25,000 IU per day of VIT-D for 4 days, then 25,000 IU per week for up to 6 weeks	No hospitalizations in IG after 21 days compared to 14% in CG; no significant mortality differences
Karonova et al. (2020–2021)/Russia	Cholecalciferol	Single-center RCT	56 IG, 54 CG	Cholecalciferol at 50,000 IU on first and eighth days of hospitalization	IG showed higher neutrophil and lymphocyte counts, lower C-RP level on ninth day of hospitalization
Majidi et al. (2020)/Iran	VIT-C	Single-center RCT	31 IG, 69 CG	One capsule of 500 mg of VIT-C daily for 14 days	Higher survival rate in IG (*p* = 0.028)
Beigmohammadi et al. (2020)/Iran	Multi-vitamins (A-B-C-D-E)	Single-center RCT	30 IG, 30 CG	25,000 IU daily of vitamins A, 600,000 IU once during study of VIT-D, 300 IU twice daily of VIT-E, 500 mg four times daily of VIT-C, Vit-B complex for 7 days	Significant improvements in serum levels of vitamins, ESR, C-RP, IL6, TNF-a, and SOFA score after intervention
Doaei et al. (2020)/Iran	Omega-3	Single-center RCT	28 IG, 73 CG	1000 mg omega-3 daily containing 400 mg EPA and 200 mg DHA added in Enteral Formula for 2 weeks after ICU admission	Higher 1-month survival rate, arterial pH levels, bicarbonate, and base excess in IG compared to CG
Gutiérrez-Castrellón et al. (2020)/Mexico	Probiotics	Single-center RCT	147 IG, 146 CG	Strains *Lactiplantibacillus plantarum* KABP022, KABP023, KAPB033, *Pediococcus acidilactici* KABP021 (totaling 2 × 10^9^ CFU)	Complete symptomatic remission and viral clearance at day 30 higher in IG [RR: 1.89 (95% CI 1.40–2.55); *p* < 0.001]
Bhutani et al. (2020)/USA	Lifestyle eating behaviors	Cross-sectional study	727	Online survey	Mean body weight gain of 0.62 kg during lockdown, increased BMI (*p* < 0.01)
Skotnicka et al. (2020)/Poland-Austria-United Kingdom	Lifestyle eating behaviors	Retrospective observational study	1071	Online survey	Increased frequency of eating, ordering ready meals, eating sweets, fruits, and drinking alcohol; decreased physical activity, increased body mass
Cicero et al. (2020)/Italy	Lifestyle eating behaviors	Cross-sectional study	359	Phone interview	No significant changes in lifestyle or BMI (*p* = 0.361)
Mascherini et al. (2020)/Italy	Lifestyle eating behaviors	Cross-sectional study	1383	Online survey	Increase in body weight from 64.9 ± 13.8 to 65.3 ± 14.1 kg (*p* < 0.001)
Chin et al. (2020)/Malaysia	Lifestyle eating behaviors	Cross-sectional study	1319	Online survey	41.2% felt eating patterns were healthier, 36.3% reduced physical activities, 25.7% had lower sleep quality
Paltrinieri et al. (2020)/Italy	Lifestyle eating behaviors	Cross-sectional study	1826	Online survey	Working remotely or in usual modalities positively influenced lifestyle, reducing likelihood of worsening physical activity (OR 0.50; 95% CI 0.31–0.79)

Legend. IG: intervention group; CG: control group; RCT: randomized controlled trial; IU: international units; VIT-C: vitamin C; VIT-D: vitamin D; VIT-E: vitamin E; GZ: gluconate zinc; DMB: vitamin D, magnesium, and B12; 25OH-D: 25-hydroxyvitamin D; C-RP: C-reactive protein; IL6: interleukin 6; TNF-a: tumor necrosis factor-alpha; ICU: intensive care unit; RR: relative risk; CI: confidence interval; SOFA: sequential organ failure assessment; EPA: eicosapentaenoic acid; DHA: docosahexaenoic acid; CFU: colony-forming units; BMI: body mass index; OR: odds ratio.

## Supplementary Materials

The following supporting information can be downloaded at: https://www.mdpi.com/article/10.3390/diseases12080193/s1, File S1: Search strategy.

## References

[1] World Health Organization (WHO) Coronavirus. https://www.who.int/health-topics/coronavirus.

[2] COVID-19 Coronavirus Pandemic COVID-19. https://www.worldometers.info/coronavirus/.

[3] Maioli C., Cioni F., Ciappellano S. (2021). COVID-19 and Nutrition Implications: A Review. Prog. Nutr..

[4] Demirci O.O. (2023). Eating disorder and its relationship with psychological distress in the COVID-19 pandemic in Turkey. Prog. Nutr..

[5] Pandey V., Mohan R., Kumar A., Gangadevi P., Kurien N. (2023). The Impact of the COVID-19 Outbreak on Lifestyle-Related Behavior Among the General Population. Cureus.

[6] Lai H., Yang M., Sun M., Pan B., Wang Q., Wang J., Tian J., Ding G., Yang K., Song X. (2022). Risk of incident diabetes after COVID-19 infection: A systematic review and meta-analysis. Metabolism.

[7] Luo W., Liu X., Bao K., Huang C. (2022). Ischemic stroke associated with COVID-19: A systematic review and meta-analysis. J. Neurol..

[8] Garg A., Posa M.K., Kumar A. (2023). Diabetes and deaths of COVID-19 patients: Systematic review of meta-analyses. Health Sci. Rev..

[9] Palaiodimos L., Kokkinidis D.G., Li W., Karamanis D., Ognibene J., Arora S., Southern W.N., Mantzoros C.S. (2020). Severe obesity, increasing age and male sex are independently associated with worse in-hospital outcomes, and higher in-hospital mortality, in a cohort of patients with COVID-19 in the Bronx, New York. Metabolism.

[10] Kruglikov I.L., Scherer P.E. (2020). The Role of Adipocytes and Adipocyte-Like Cells in the Severity of COVID-19 Infections. Obesity.

[11] Zhou Y., Chi J., Lv W., Wang Y. (2021). Obesity and diabetes as high-risk factors for severe coronavirus disease 2019 (COVID-19). Diabetes Metab. Res. Rev..

[12] Obukhov A.G., Stevens B.R., Prasad R., Li Calzi S., Boulton M.E., Raizada M.K., Oudit G.Y., Grant M.B. (2020). SARS-CoV-2 Infections and ACE2: Clinical Outcomes Linked with Increased Morbidity and Mortality in Individuals with Diabetes. Diabetes.

[13] Lee M.M.Y., Docherty K.F., Sattar N., Mehta N., Kalra A., Nowacki A.S., Solomon S.D., Vaduganathan M., Petrie M.C., Jhund P.S. (2022). Renin-angiotensin system blockers, risk of SARS-CoV-2 infection and outcomes from COVID-19: Systematic review and meta-analysis. Eur. Heart J. Cardiovasc. Pharmacother..

[14] Giryes S., Bragazzi N.L., Bridgewood C., De Marco G., McGonagle D. (2022). COVID-19 Vasculitis and vasculopathy-Distinct immunopathology emerging from the close juxtaposition of Type II Pneumocytes and Pulmonary Endothelial Cells. Semin. Immunopathol..

[15] Lee H.W., Yoon C.H., Jang E.J., Lee C.H. (2021). Renin-angiotensin system blocker and outcomes of COVID-19: A systematic review and meta-analysis. Thorax.

[16] Jacks R.D., Lumeng C.N. (2024). Macrophage and T cell networks in adipose tissue. Nat. Rev. Endocrinol..

[17] Misumi I., Starmer J., Uchimura T., Beck M.A., Magnuson T., Whitmire J.K. (2019). Obesity Expands a Distinct Population of T Cells in Adipose Tissue and Increases Vulnerability to Infection. Cell Rep..

[18] Karagiannis F., Peukert K., Suracey L., Michla M., Nikolka F., Fox M. (2022). Impaired ketogenesis ties metabolism to T cell dysfunction in COVID-19. Nature.

[19] Detopoulou P., Demopoulos C.A., Antonopoulou S. (2021). Micronutrients, Phytochemicals and Mediterranean Diet: A Potential Protective Role against COVID-19 through Modulation of PAF Actions and Metabolism. Nutrients.

[20] Center for Disease Control and Prevention (CDC) Underlying Medical Conditions Associated with Higher Risk for Severe COVID-19: Information for Healthcare Professionals. https://archive.cdc.gov/#/details?url=https://www.cdc.gov/coronavirus/2019-ncov/hcp/clinical-care/underlyingconditions.html.

[21] Renata R.N., Arely G.A., Gabriela L.A., Esther M.M. (2023). Immunomodulatory Role of Microelements in COVID-19 Outcome: A Relationship with Nutritional Status. Biol. Trace Elem. Res..

[22] Rust P., Ekmekcioglu C. (2023). The Role of Diet and Specific Nutrients during the COVID-19 Pandemic: What Have We Learned over the Last Three Years?. Int. J. Environ. Res. Public Health.

[23] Amir-Behghadami M., Janati A. (2020). Population, Intervention, Comparison, Outcomes and Study (PICOS) design as a framework to formulate eligibility criteria in systematic reviews. Emerg. Med. J..

[24] Sabico S., Enani M.A., Sheshah E., Aljohani N.J., Aldisi D.A., Alotaibi N.H., Alshingetti N., Alomar S.Y., Alnaami A.M., Amer O.E. (2021). Effects of a 2-Week 5000 IU versus 1000 IU Vitamin D3 Supplementation on Recovery of Symptoms in Patients with Mild to Moderate COVID-19: A Randomized Clinical Trial. Nutrients.

[25] Murai I.H., Fernandes A.L., Sales L.P., Pinto A.J., Goessler K.F., Duran C.S.C., Silva C.B.R., Franco A.S., Macedo M.B., Dalmolin H.H. (2021). Effect of a Single High Dose of Vitamin D3 on Hospital Length of Stay in Patients With Moderate to Severe COVID-19: A Randomized Clinical Trial. JAMA.

[26] Thomas S., Patel D., Bittel B., Wolski K., Wang Q., Kumar A., Il’Giovine Z.J., Mehra R., McWilliams C., Nissen S.E. (2021). Effect of High-Dose Zinc and Ascorbic Acid Supplementation vs Usual Care on Symptom Length and Reduction Among Ambulatory Patients With SARS-CoV-2 Infection: The COVID A to Z Randomized Clinical Trial. JAMA Netw. Open.

[27] Maghbooli Z., Sahraian M.A., Jamalimoghadamsiahkali S., Asadi A., Zarei A., Zendehdel A., Varzandi T., Mohammadnabi S., Alijani N., Karimi M. (2021). Treatment with 25-Hydroxyvitamin D3 (Calcifediol) Is Associated with a Reduction in the Blood Neutrophil-to-Lymphocyte Ratio Marker of Disease Severity in Hospitalized Patients with COVID-19: A Pilot Multicenter, Randomized, Placebo-Controlled, Double-Blinded Clinical Trial. Endocr. Pract..

[28] Annweiler C., Beaudenon M., Gautier J., Gonsard J., Boucher S., Chapelet G., Darsonval A., Fougère B., Guérin O., Houvet M. (2022). High-dose versus standard-dose vitamin D supplementation in older adults with COVID-19 (COVIT-TRIAL): A multicenter, open-label, randomized controlled superiority trial. PLoS Med..

[29] Mariani J., Antonietti L., Tajer C., Ferder L., Inserra F., Sanchez Cunto M., Brosio D., Ross F., Zylberman M., López D.E. (2022). High-dose vitamin D versus placebo to prevent complications in COVID-19 patients: Multicentre randomized controlled clinical trial. PLoS ONE.

[30] De Niet S., Trémège M., Coffiner M., Rousseau A.F., Calmes D., Frix A.N., Gester F., Delvaux M., Dive A.F., Guglielmi E. (2022). Positive Effects of Vitamin D Supplementation in Patients Hospitalized for COVID-19: A Randomized, Double-Blind, Placebo-Controlled Trial. Nutrients.

[31] Karonova T.L., Golovatyuk K.A., Kudryavtsev I.V., Chernikova A.T., Mikhaylova A.A., Aquino A.D., Lagutina D.I., Zaikova E.K., Kalinina O.V., Golovkin A.S. (2022). Effect of Cholecalciferol Supplementation on the Clinical Features and Inflammatory Markers in Hospitalized COVID-19 Patients: A Randomized, Open-Label, Single-Center Study. Nutrients.

[32] Majidi N., Rabbani F., Gholami S., Gholamalizadeh M., BourBour F., Rastgoo S., Hajipour A., Shadnoosh M., Akbari M.E., Bahar B. (2021). The Effect of Vitamin C on Pathological Parameters and Survival Duration of Critically Ill Coronavirus Disease 2019 Patients: A Randomized Clinical Trial. Front. Immunol..

[33] Beigmohammadi M.T., Bitarafan S., Hoseindokht A., Abdollahi A., Amoozadeh L., Soltani D. (2021). The effect of supplementation with vitamins A, B, C, D, and E on disease severity and inflammatory responses in patients with COVID-19: A randomized clinical trial. Trials.

[34] Doaei S., Gholami S., Rastgoo S., Gholamalizadeh M., Bourbour F., Bagheri S.E., Samipoor F., Akbari M.E., Shadnoush M., Ghorat F. (2021). The effect of omega-3 fatty acid supplementation on clinical and biochemical parameters of critically ill patients with COVID-19: A randomized clinical trial. J. Transl. Med..

[35] Gutiérrez-Castrellón P., Gandara-Martí T., Abreu YAbreu A.T., Nieto-Rufino C.D., López-Orduña E., Jiménez-Escobar I., Jiménez-Gutiérrez C., López-Velazquez G., Espadaler-Mazo J. (2022). Probiotic improves symptomatic and viral clearance in COVID-19 outpatients: A randomized, quadruple-blinded, placebo-controlled trial. Gut Microbes.

[36] Tan C.W., Ho L.P., Kalimuddin S., Cherng B.P.Z., Teh Y.E., Thien S.Y., Wong H.M., Tern P.J.W., Chandran M., Chay J.W.M. (2020). Cohort study to evaluate the effect of vitamin D, magnesium, and vitamin B12 in combination on progression to severe outcomes in older patients with coronavirus (COVID-19). Nutrition.

[37] Skotnicka M., Karwowska K., Kłobukowski F., Wasilewska E., Małgorzewicz S. (2021). Dietary Habits before and during the COVID-19 Epidemic in Selected European Countries. Nutrients.

[38] Caballero-García A., Pérez-Valdecantos D., Guallar P., Caballero-Castillo A., Roche E., Noriega D.C., Córdova A. (2021). Effect of Vitamin D Supplementation on Muscle Status in Old Patients Recovering from COVID-19 Infection. Medicina.

[39] Bhutani S., vanDellen M.R., Cooper J.A. (2021). Longitudinal Weight Gain and Related Risk Behaviors during the COVID-19 Pandemic in Adults in the US. Nutrients.

[40] Cicero A.F.G., Fogacci F., Giovannini M., Mezzadri M., Grandi E., Borghi C. (2021). COVID-19-Related Quarantine Effect on Dietary Habits in a Northern Italian Rural Population: Data from the Brisighella Heart Study. Nutrients.

[41] Mascherini G., Catelan D., Pellegrini-Giampietro D.E., Petri C., Scaletti C., Gulisano M. (2021). Changes in physical activity levels, eating habits and psychological well-being during the Italian COVID-19 pandemic lockdown: Impact of socio-demographic factors on the Florentine academic population. PLoS ONE.

[42] Chin Y.S., Woon F.C., Chan Y.M. (2022). The impact of Movement Control Order during the COVID-19 pandemic on lifestyle behaviours and body weight changes: Findings from the MyNutriLifeCOVID-19 online survey. PLoS ONE.

[43] Paltrinieri S., Bressi B., Costi S., Mazzini E., Cavuto S., Ottone M., De Panfilis L., Fugazzaro S., Rondini E., Rossi P.G. (2021). Beyond Lockdown: The Potential Side Effects of the SARS-CoV-2 Pandemic on Public Health. Nutrients.

[44] Rastogi A., Bhansali A., Khare N., Suri V., Yaddanapudi N., Sachdeva N., Puri G.D., Malhotra P. (2022). Short term, high-dose vitamin D supplementation for COVID-19 disease: A randomised, placebo-controlled, study (SHADE study). Postgrad. Med. J..

[45] Cangelosi G., Grappasonni I., Nguyen C.T.T., Acito M., Pantanetti P., Benni A., Petrelli F. (2023). Mediterranean Diet (MedDiet) and Lifestyle Medicine (LM) for support and care of patients with type II diabetes in the COVID-19 era: A cross-observational study. Acta Biomed..

[46] Nirala S.K., Naik B.N., Rao R., Pandey S., Singh C.M., Chaudhary N. (2022). Impact of Lockdown due to COVID-19 on lifestyle and diet pattern of college students of Eastern India: A cross-sectional survey. Nepal J. Epidemiol..

[47] Cangelosi G., Acito M., Grappasonni I., Nguyen C.T.T., Tesauro M., Pantanetti P., Morichetti L., Ceroni E., Benni A., Petrelli F. (2024). Yoga or Mindfulness on Diabetes: Scoping Review for Theoretical Experimental Framework. Ann. Ig..

[48] Argano C., Mallaci Bocchio R., Natoli G., Scibetta S., Lo Monaco M., Corrao S. (2023). Protective Effect of Vitamin D Supplementation on COVID-19-Related Intensive Care Hospitalization and Mortality: Definitive Evidence from Meta-Analysis and Trial Sequential Analysis. Pharmaceuticals.

[49] Lai Y.H., Fang T.C. (2013). The pleiotropic effect of vitamin d. ISRN Nephrol..

[50] Taha R., Abureesh S., Alghamdi S., Hassan R.Y., Cheikh M.M., Bagabir R.A., Almoallim H., Abdulkhaliq A. (2021). The Relationship Between Vitamin D and Infections Including COVID-19: Any Hopes?. Int. J. Gen. Med..

[51] Ashique S., Gupta K., Gupta G., Mishra N., Singh S.K., Wadhwa S., Gulati M., Dureja H., Zacconi F., Oliver B.G. (2023). Vitamin D-A prominent immunomodulator to prevent COVID-19 infection. Int. J. Rheum. Dis..

[52] Louca P., Murray B., Klaser K., Graham M.S., Mazidi M., Leeming E.R., Thompson E., Bowyer R., Drew D.A., Nguyen L.H. (2021). Modest effects of dietary supplements during the COVID-19 pandemic: Insights from 445,850 users of the COVID-19 Symptom Study app. BMJ Nutr. Prev. Health.

[53] Mancin S., Mazzoleni B. (2023). Probiotics as adjuvant therapy in the treatment of Allergic Rhinitis. Res. J. Pharm. Technol..

[54] Kumar M., Pal N., Sharma P., Kumawat M., Sarma D.K., Nabi B., Verma V., Tiwari R.R., Shubham S., Arjmandi B. (2022). Omega-3 Fatty Acids and Their Interaction with the Gut Microbiome in the Prevention and Amelioration of Type-2 Diabetes. Nutrients.

