# Supplementary material for: COVID-19, Nutrients and Lifestyle Eating Behaviors: A Narrative Review
Source: Diseases. 2024 Aug 22;12(8):193. doi: 10.3390/diseases12080193 (PMC11354052; doi:10.3390/diseases12080193)
Supplement: Supplementary file 1 [file diseases-12-00193-s001.zip › diseases-3116894-supplementary.pdf]

File S1. Search Strategy

(("Nutritional Sciences" OR "Nutritional Status" OR "Nutrition Therapy" OR "Nutrition Assessment" OR "Nutrition Surveys" OR "Diet, Food, and Nutrition" OR "Diet, Vegetarian" OR "Diet, Healthy") OR (("Diet" OR "Diet, Carbohydrate-Restricted" OR "Diet, Protein-Restricted" OR "Diet, Fat-Restricted" OR "Diet, Macrobiotic" OR "Diet, Vegetarian") OR ("Diet, Diabetic" OR "Diet, High-Protein Low-Carbohydrate" OR "Diet, High-Protein" OR "Diet, Vegan" OR "Diet, Food, and Nutrition" OR "Caloric Restriction" OR "Feeding Behaviour"))) AND (("COVID-19" OR "SARS-COV-2") OR (COVID)) NOT review Filters: Humans, English, from 1000/1/1 - 2023/10/31	**Results**: 1943
